# Altered Electrical, Biomolecular, and Immunologic Phenotypes in a Novel Patient-Derived Stem Cell Model of Desmoglein-2 Mutant ARVC

**DOI:** 10.3390/jcm10143061

**Published:** 2021-07-10

**Authors:** Robert N. Hawthorne, Adriana Blazeski, Justin Lowenthal, Suraj Kannan, Roald Teuben, Deborah DiSilvestre, Justin Morrissette-McAlmon, Jeffrey E. Saffitz, Kenneth R. Boheler, Cynthia A. James, Stephen P. Chelko, Gordon Tomaselli, Leslie Tung

**Affiliations:** 1 Department of Biomedical Engineering, School of Medicine, Johns Hopkins University, Baltimore, MD 21205, USA; rhawtho4@jhmi.edu (R.N.H.); ablazesk@mit.edu (A.B.); jilowenthal@jhmi.edu (J.L.); skannan4@jhmi.edu (S.K.); rteuben1@jhmi.edu (R.T.); jmorri65@jhmi.edu (J.M.-M.); kbohele1@jhmi.edu (K.R.B.); 2 Medical Scientist Training Program, School of Medicine, Johns Hopkins University, Baltimore, MD 21205, USA; 3 Department of Medicine, School of Medicine, Johns Hopkins University, Baltimore, MD 21205, USA; ddisilvestre@som.umaryland.edu (D.D.); cjames7@jhmi.edu (C.A.J.); 4 Department of Pathology, Beth Israel Deaconess Medical Center, Boston, MA 02215, USA; jsaffitz@bidmc.harvard.edu; 5 Department of Biomedical Sciences, College of Medicine, Florida State University, Tallahassee, FL 32306, USA; 6 Department of Medicine, Albert Einstein College of Medicine, Bronx, NY 10461, USA

**Keywords:** arrhythmogenic cardiomyopathy, arrhythmogenic right ventricular cardiomyopathy, ARVC, desmoglein-2, NFκB signaling, patient-derived stem cells, induced pluripotent stem cells

## Abstract

Arrhythmogenic right ventricular cardiomyopathy (ARVC) is a progressive heart condition which causes fibro-fatty myocardial scarring, ventricular arrhythmias, and sudden cardiac death. Most cases of ARVC can be linked to pathogenic mutations in the cardiac desmosome, but the pathophysiology is not well understood, particularly in early phases when arrhythmias can develop prior to structural changes. Here, we created a novel human induced pluripotent stem cell-derived cardiomyocyte (hiPSC-CM) model of ARVC from a patient with a c.2358delA variant in desmoglein-2 (*DSG2*). These *DSG2*-mutant (*DSG2*^Mut^) hiPSC-CMs were compared against two wildtype hiPSC-CM lines via immunostaining, RT-qPCR, Western blot, RNA-Seq, cytokine expression and optical mapping. Mutant cells expressed reduced *DSG2* mRNA and had altered localization of desmoglein-2 protein alongside thinner, more disorganized myofibrils. No major changes in other desmosomal proteins were noted. There was increased pro-inflammatory cytokine expression that may be linked to canonical and non-canonical NFκB signaling. Action potentials in *DSG2*^Mut^ CMs were shorter with increased upstroke heterogeneity, while time-to-peak calcium and calcium decay rate were reduced. These were accompanied by changes in ion channel and calcium handling gene expression. Lastly, suppressing *DSG2* in control lines via siRNA allowed partial recapitulation of electrical anomalies noted in *DSG2*^Mut^ cells. In conclusion, the aberrant cytoskeletal organization, cytokine expression, and electrophysiology found *DSG2*^Mut^ hiPSC-CMs could underlie early mechanisms of disease manifestation in ARVC patients.

## 1. Introduction

Arrhythmogenic right ventricular cardiomyopathy (ARVC) is an inherited, progressive arrhythmogenic cardiomyopathy (ACM) characterized by cardiomyocyte death and fibro-fatty scarring in the ventricular myocardium. Clinically, patients with ARVC develop ventricular arrhythmias which often present more frequently as the disease progresses. ARVC is estimated to account for more than 10% of all cardiovascular deaths in patients younger than 65 years old and is a leading cause of sudden cardiac death (SCD) in young athletes [1,2,3]. ARVC prevalence is approximately 1:1000–1:5000, and patients are typically diagnosed in young adulthood; diagnosis before puberty is extremely rare [4,5,6].

While the pathophysiology underlying ARVC is still poorly understood, there has been general scientific consensus that the common final pathway of the disease involves the disruption of the cardiac desmosome, a cellular structure present at the intercalated disc that is critical for intercellular mechanical and electrical coupling between cardiomyocytes [7,8]. Pathogenic variants in any of the five cardiac desmosomal genes – plakoglobin (*JUP)*, plakophilin-2 (*PKP2*), desmoglein-2 (*DSG2*), desmoplakin (*DSP*) and desmocollin-2 (*DSC2*)—account for more than 60% of inherited ARVC and have a combined estimated prevalence of approximately 1.2 per 1000 in the general population [9]. Among these, *PKP2* and *DSG2* are the first and second most common variants, respectively [6,10]. 

Determining the pathogenic mechanisms underlying ARVC has proven difficult. The study of human tissue is challenging, as ARVC is frequently diagnosed in later stages of disease (e.g., myocardial scarring) when retrieval of a myocardial biopsy presents significant risk of cardiac perforation. And while patients with advanced ARVC develop severe myocardial scarring and cardiomyocyte loss, which can contribute to arrhythmic risk, substantial evidence demonstrates that cardiac electrical abnormalities and elevated risk of dangerous arrhythmias—develop well before any overt structural deterioration can be detected [11,12,13]. Several animal models of ARVC have been established, including in mice [14,15,16,17] and dogs [18,19], which have proven useful in studying specific pathophysiologic processes, but these models are limited due to substantial differences in cardiac electrophysiology between animals and humans [20]. Human induced pluripotent stem cell-derived cardiomyocytes (hiPSC-CMs) present a powerful model for studying cardiac disease, and ARVC patient-specific hiPSC-CMs have been shown to recapitulate key characteristics observed in human disease [21]. By generating and studying hiPSC-CMs harboring unique pathogenic variants associated with ARVC, we can begin to uncover the mechanisms by which diseased cardiomyocytes behave differently in this early “concealed phase” of ARVC, revealing new potential targets for therapeutic intervention.

Prior studies show that *PKP2*-mutant hiPSC-CMs display reduced membrane localization of plakophilin-2 and plakoglobin, in conjunction with disrupted desmosomal structure [22,23]. These models have also revealed a number of electrical defects. PKP2-mutant hiPSC-CM models display altered calcium homeostasis and connexin-43 function [17] while both *PKP2-* and *DSG2*-mutant hiPSC-CM models demonstrated slower action potential upstrokes and reduced sodium current density [24,25]. Inflammatory infiltration by immune cells has been shown to play an important role in pathologic remodeling of the myocardium in ARVC, and recent studies have indicated that ARVC hiPSC-CMs produce and secrete inflammatory cytokines via innate activation of nuclear factor-κB (NFκB) [15]. In *Dsg2*-mutant mice, inhibition of NFκB via the small molecule antagonist, Bay11-7082, prevented myocardial NFκB-mediated cytokine storm and inflammatory infiltration.

In this study, we developed a novel hiPSC-CM model of ARVC derived from a patient with a likely pathogenic variant in *DSG2*. We characterized a variety of functional and molecular features that could help link the cellular behavior with clinical manifestation of ARVC. Our findings show that hiPSC-CMs harboring this mutation display altered electrophysiological properties and augmented immune cytokine expression, even when compared to ARVC hiPSC-CMs reported in other studies, suggesting that significant phenotypic variation exists among models with different causative variants.

## 2. Materials and Methods

### 2.1. hiPSC Line Generation

Blood samples were collected from the donor (JHU013), who met ‘definite’ 2010 Task Force Criteria for diagnosis of ARVC [26]. Peripheral blood mononuclear cells (PBMCs) were isolated and reprogrammed into pluripotency via Sendai-viral transfection of the Yamanaka factors (see Supplementary Methods for complete protocol). Clonal colonies were selected and expanded until passage 10. DNA was then isolated to verify the loss of the Sendai virus and clones were screened via Sanger sequencing and karyotyping.

### 2.2. hiPSC-CM Differentiation and Culture

In addition to the *DSG2* c.2358delA hiPSC line generated, we used two control lines. One was generated from an age- and sex-matched donor under identical protocols (JHU001, “Ctrl1”) [27,28] and the other is a widely used wildtype control line kindly gifted to us by Dr. Bruce Conklin at the University of California, San Francisco (WTC11, “Ctrl2”) [29], which can be obtained from the NIGMS Human Genetic Cell Repository at the Coriell Institute for Medical Research: GM25256. 

For the detailed differentiation protocol please refer to Supplementary Methods. Briefly, hiPSCs were subjected to cardiogenic differentiation via previously described methods using small molecule modulation of Wnt signaling [30,31]. Spontaneous beating was observed around day 7–9. Beginning between days 10–14, hiPSC-CM cultures underwent 4-day purification in glucose-depleted DMEM supplemented with 4 mM L-lactate (lactate medium; MilliporeSigma, St. Louis, MO, USA). On differentiation day 28, hiPSC-CMs were re-plated in RPMI1640 plus B27 supplement (B27+ media; Thermo Fisher, Waltham, MA, USA) onto Geltrex-coated Thermanox coverslips (Thermo Fisher) at a density of 250,000 cells/cm^2^. hiPSC-CM monolayers were then cultured for 7–10 days, with media changes every other day, until evaluation. 

### 2.3. Biomolecular Characterization Using Immunostaining, Real-Time Quantitative PCR, Western Blot, and Cytokine Arrays

For immunostaining, cells were fixed in 4% paraformaldehyde, and probed using standard immunocytochemistry techniques. hiPSCs were stained for OCT3/4, SOX2, and Nanog, and hiPSC-CM monolayers were stained for desmoglein-2, plakoglobin, plakophilin-2, cardiac troponin I, α-actinin and nuclei (see Table S3 for primary antibody details). Images were acquired via confocal microscopy (ZEISS, Oberkochen, Germany). To assess Z-line length in hiPSC-CMs, 20 µm x 20 µm regions of interest were selected from high magnification images at random by computer script, and α-actinin-immunopositive lines were measured by a blinded observer using image analysis software (ImageJ, Version 2.0.0-rc-69/1.53c).

For RT-qPCR experiments, total RNA was extracted from hiPSC-CMs on differentiation day 37–39 using a column preparation (RNeasy Mini Kit; Qiagen Sciences, Germantown, MD, USA) and reverse transcription was performed using the High-Capacity cDNA Reverse Transcription Kit (Applied Biosystems, Waltham, MA, USA). cDNA was amplified using PowerUp SYBR Green PCR master mix (Applied Biosystems) and a QuantStudio 7 Flex RT-qPCR system (Applied Biosystems) under manufacturer’s recommended protocol. All assays were performed with three technical replicates and a minimum of three pooled cell samples. GAPDH and 18S rRNA were used in combination as internal controls; all primer sequences are listed in Table S1.

For Western blots, total protein was extracted from hiPSC-CMs using RIPA lysis buffer (Cell Signaling, Danvers, MA, USA). Samples were prepared and run under denaturing conditions on NuPAGE 4–12% Bis-Tris gels in MOPS buffer using manufacturers recommended reagents and protocol (Thermo Fisher). Proteins were transferred to PVDF membranes using the Trans-Blot Turbo system (Bio-Rad Laboratories, Hercules, CA, USA), probed with primary antibodies (see Table S3) and visualized with fluorescent secondary antibodies (LI-COR, Lincoln, NE, USA). Images were obtained and analyzed using an Odyssey CLx imaging system (LI-COR).

For cytokine analysis, supernatant was aspirated from day 37 hiPSC-CMs, two days after feeding and flash frozen until analysis. Then, hiPSC-CMs were isolated and lysed in RIPA buffer as described above. Cell lysates and cell culture supernatants were analyzed using the Proteome Profiler Human XL Cytokine Array Kit (R&D Systems, Minneapolis, MN, USA), and films were scanned and analyzed using Quick Spots image analysis software (Version 25.0.0r, Western Vision Software, Salt Lake City, UT, USA). 

### 2.4. RNA Sequencing and Analysis

hiPSC-CMs cultured to differentiation day 35 were gently dislodged with a cell scraper (Thermo Fisher), suspended in TRIzol reagent and flash frozen for storage. Isolated RNA across the samples had a mean RIN of 9.2. RNA-Seq libraries were prepared by an outside vendor (GENEWIZ, South Plainfield, NJ, USA) using a standard poly-A selection protocol for isolating mRNAs. Final libraries were sequenced as 2x150 bp paired-end sequencing on an Illumina HiSeq. Samples were sequenced to a depth of 30–40 million paired reads/cell. Libraries were mapped to the human genome (GRCh38) using STAR (2.3.1a) [32] with two-pass mode, and then counted using featureCounts (2.0.0) through the Subread package (Version 2.0.2) [33,34]. Identification of differentially expressed genes was done using DESeq2 [35], with genes identified as differentially expressed by Benjamini-Hochberg-adjusted *p*-value < 0.05. KEGG pathway maps were made using the Pathview package (Version 1.26.0) [36]. 

### 2.5. Optical Mapping of Calcium and Transmembrane Potential

All staining and mapping steps were performed in Tyrode’s solution (see Supplementary Methods) containing 10 µM blebbistatin (PeproTech, Cranbury, NJ, USA) to suppress contraction and prevent motion artifact. Coverslips with hiPSC-CM monolayers were stained with either the voltage indicator di-4-ANEPPS (10 µM, 10 min; Thermo Fisher) or calcium indicator Rhod-2 AM (1 µM, 15 min; Thermo Fisher); to aid cell loading, staining solutions were supplemented with 0.05% Pluronic F-127. Coverslips were then gently washed and transferred to a 35 mm petri dish with fresh mapping solution. The dish was placed on a stage heated to 37 °C for the remainder of the experiment. hiPSC-CM monolayers were stimulated from one edge with a palladium line electrode delivering 10 V, 10 ms monophasic rectangular pulses at predetermined cycle lengths: 1000 ms, 700 ms, and 500 ms. Optical mapping recordings were taken using a 100x100 pixel CMOS camera (MiCAM Ultima-L; SciMedia, Costa Mesa, CA, USA) and analyzed using custom MATLAB scripts (Version R2019b, MathWorks, Natick, MA, USA; details in Supplementary Methods).

### 2.6. siRNA Transfection

Pre-designed siRNA oligos targeted against *DSG2* were purchased (MilliporeSigma), and a cocktail of three distinct oligos (see Table S2 for oligo sequences) was complexed for transfection using TransIT-siQUEST transfection reagent (Mirus Bio, Madison, WI, USA). On differentiation day 35, hiPSC-CM media was replaced with B27+ media supplemented with siRNA complexes for a final concentration of 50 nM. 48 h later, the media was replaced with fresh B27+ media, and the samples were analyzed the following day (72 h total treatment time).

### 2.7. Statistics

Unless otherwise indicated, all data are presented as mean ± 95% confidence interval, calculated in R. Continuous variables were compared using unpaired, unequal variance Student’s *t*-test with a Holm-Bonferroni family-wise error correction. *p* < 0.05 in two-tailed analysis was considered significant. For RT-qPCR data, normalized relative quantities and their standard errors were calculated using the EasyqpcR software package (Version 1.34) [37].

## 3. Results

### 3.1. Family History and Clinical Phenotypes of ARVC Patient Donor

**Figure 1:**
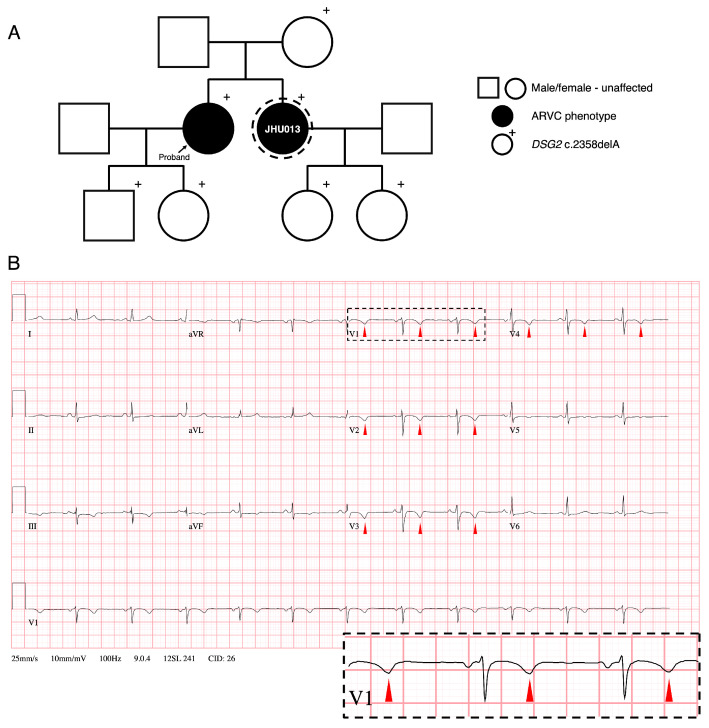
Family pedigree and electrocardiogram from hiPSC donor. (**A**) Family pedigree of patient JHU013 (dashed circle) harboring a pathogenic variant in desmoglein-2 (*DSG2* c.2358delA). Arrow signifies proband. (**B**) 12-lead ECG of patient JHU013 showing T wave inversion in leads V1-V4 (red markers), a classic feature in ARVC patients. Dashed box inset, highlighting T wave inversion in lead V1.

The patient (identified as JHU013) is a 48-year-old woman of South Asian descent who harbors a familial genetic variant in *DSG2* (Figure 1A). Her 12-lead electrocardiogram (ECG) was positive for T wave inversions (initially observed at age 22; Figure 1B) and consistently negative for epsilon waves. She began experiencing paroxysmal dizziness and palpitations at age 32, but she was not diagnosed with ARVC until age 45 when a diagnosis in her sister led to a thorough cardiac evaluation. Specifically, her ECG showed T wave inversions in anterior precordial leads V1-V4 with inferior repolarization abnormalities in leads II, III, and aVF (Figure 1B, Table 1). Cardiac magnetic resonance (CMR) imaging demonstrated mild dilation of the right ventricle and dyskinesia of the right ventricular free wall (108 mL/m^2^ RV end-diastolic volume to body surface area; Table 1, Figure S1) with microaneurysm formation along the right ventricular outflow tract. CMR was also notable for extensive delayed gadolinium enhancement involving the right ventricular free and inferior walls, suggesting significant fibrosis in those regions. However, no ventricular wall fat infiltration was observed. As of last follow-up, the hiPSC donor had no documented history of sustained ventricular arrhythmia. Further familial screening revealed several carriers of the *DSG2* c.2358delA variant, including the donor, her sister (proband), nephew, niece, her mother, and both of her children (Figure 1A). At last follow-up, none of the children of the donor or her sister who carry the variant (ages 13–20) had been diagnosed with ARVC, though they are on the low end of the age range at which symptoms typically present. However, the younger family members have experienced some notable cardiac abnormalities. A Holter monitor revealed 420 premature ventricular complexes (PVCs) in the proband’s son (age 20) over a 24-hour period, which was high but does not reach the 2010 Task Force criteria of ≥500 PVCs over 24 h. The proband’s daughter (age 17) has a history of palpitations, and one of the donor’s daughters (age 16) has a history of palpitations as well as T wave inversions in V1 and V3 on ECG.

### 3.2. Establishment of Patient-Specific Human Induced Pluripotent Stem Cell Line with Novel Dsg2 Pathogenic Variant

**Figure 2:**
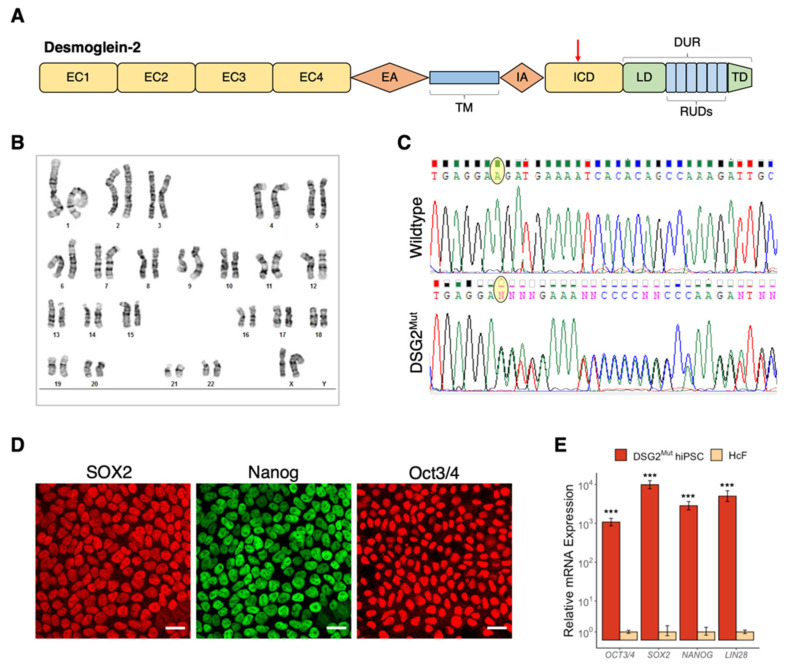
Donor-derived hiPSC line harbors heterozygous *DSG2* c.2358delA variant and expresses markers of pluripotency. (**A**) Schematic protein structure of desmoglein-2. EC1–4, extracellular cadherin domains; EA, extracellular anchor domain; TM, transmembrane domain; IA, intracellular anchor domain; ICD, intracellular cadherin-like domain; LD, linker domain; RUD, repeat unit domain with 6 repeats; TD, terminal domain; DUR, DSG unique region; red arrow indicates location of truncation caused by the *DSG2* c.2358delA variant. (**B**) Chromosomal analysis of JHU013 (*DSG2*^Mut^) hiPSCs. (**C**) Sanger sequencing of *DSG2* in control and *DSG2*^Mut^ hiPSCs showing the c.2358delA mutation. Yellow oval highlights c.2358 nucleotide under investigation. ‘N’ indicates likely heterozygosity at that location. (**D**) Confocal images of *DSG2*^Mut^ hiPSCs depicting immunopositivity for canonical markers of pluripotency: Sex determining region Y-box 2 (SOX2), homeobox NANOG, and Octamer binding transcription factor-3/4 (OCT3/4). (**E**) RT-qPCR analysis showed abundant expression of pluripotency-associated transcripts in *DSG2*^Mut^ hiPSCs relative to terminally differentiated control cells (primary neonatal human cardiac fibroblasts, HcF). *n* = 3 for each group. *** *p* < 0.001.

Sequence analysis shows that the *DSG2* variant harbored by patient JHU013 – listed in ClinVar as NM_001943.5(DSG2):c.2358del (p.Asp787fs) – causes a frameshift mutation, resulting in the formation of a premature stop codon, 21 codons downstream. This frameshift produces a truncation in the intracellular cadherin-like domain (ICD) of the DSG2 protein (Figure 2A). In line with standards of the American College of Medical Genetics and Genomics [38,39], this variant has been classified as likely pathogenic. Cascade screening and clinical criteria are consistent with the DSG2 loss of function contributing to the pathogenesis of both the proband and JHU013.

In order to determine the functional and pathological consequences of the *DSG2* c.2358delA variant, we generated an hiPSC line from the affected patient. Briefly, peripheral blood mononuclear cells were isolated from the donor’s blood, induced to form blast cells and reprogrammed into pluripotency via transfection with Sendai virus vectors containing the Yamanaka factors. These cells were expanded, and clonal colonies were selected and characterized. Chromosomal analysis of this new *DSG2*-mutant (*DSG2*^Mut^) hiPSC line demonstrated a normal karyotype, and Sanger sequencing showed a heterozygous *DSG2* c.2358delA variant present in the reprogrammed cells (Figure 2B,C). Canonical markers of pluripotency were verified via immunofluorescent signal and RT-qPCR in *DSG2*^Mut^ hiPSCs (Figure 2D,E).

### 3.3. Biomolecular Characterization of DSG2^Mut^ hiPSC-CM Model

For controls, we selected an hiPSC line derived from a healthy volunteer of the same gender and similar age following an identical protocol (JHU001, “Ctrl1”; female), as well as a widely available wildtype control line (WTC11, “Ctrl2”; male). hiPSCs from all three lines were subjected to the same Wnt-mediated cardiogenic differentiation protocol and began to beat on approximately day 7. On days 10–14 the CM population was enriched metabolically via lactate purification as previously described. Cardiomyocytes formed a syncytial monolayer after being plated onto coverslips on day 28 and were cultured until day 35–39, when they were analyzed. Differentiated hiPSC-CMs from all three lines were measured by flow cytometry to be >90% positive for cardiac troponin T on day 35, indicating high purity of cardiomyocytes, with no differences in differentiation efficiency noted between control and *DSG2*^Mut^ hiPSC lines (Figure S2). The mean spontaneous beating rates for the hiPSC-CM monolayers measured in culture was similar among all lines on differentiation day 38 (67.6 ± 5.7 beats per minute in *DSG2*^Mut^ vs. 68.4 ± 12.1 BPM in Ctrl1 and 64.8 ± 5.8 BPM in Ctrl2; *n*= 14, 12, and 12, respectively). 

**Figure 3:**
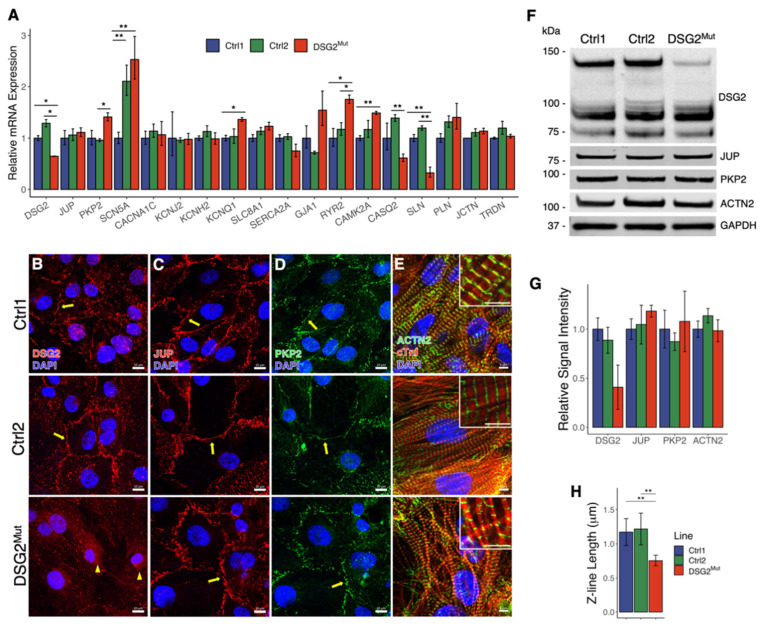
RT-qPCR, immunostaining and Western blot analyses of *DSG2*^Mut^ hiPSC-CMs. (**A**) RT-qPCR analysis of normal and *DSG2*^Mut^ hiPSC-CMs, normalized to Ctrl1. *DSG2* primer targets a region upstream of the c.2358 deletion. *n* = 3 for all groups. (**B**–**E**) Representative control and *DSG2*^Mut^ hiPSC-CMs immunostained for desmosomal and structural proteins. Yellow arrows highlight presence of desmosomal proteins at cell-cell boundaries; yellow arrowheads denote perinuclear localization of DSG2. White scale bars: 10 µm (**B**–**D**), 5 µm (**E**). (**F**) Whole lysate western immunoblots of *DSG2*^Mut^ and control hiPSC-CMs, probed for desmosomal proteins and α-actinin (ACTN2). (**G**) Quantification of western blot signal intensity relative to GAPDH and normalized to Ctrl1. DSG2 was quantified using the full-length 130 kDa band. *n* = 3 for each line. (**H**) Z-line lengths quantified from confocal images of α-actinin immunostaining. Shorter Z-lines in *DSG2*^Mut^ cells correspond to narrower myofibrils. Two regions were randomly selected for quantification from three unique images of each line. * *p* < 0.05; ** *p* < 0.01.

We first characterized the expression and localization of key desmosomal proteins and cardiac ion channels in the *DSG2*-mutant hiPSC-CM model. Specifically, RT-qPCR analysis showed that mRNA expression of *DSG2* is significantly reduced in the mutant hiPSC-CMs as compared to both Ctrl1 and Ctrl2 (35.2% and 49.6% reduction, respectively) (Figure 3A), using a primer targeted to a site upstream of the *DSG2* c.2358delA location. Sodium and potassium channel genes *SCN5A* and *KCNQ1*, respectively, were more highly expressed in *DSG2*^Mut^ CMs compared to Ctrl1, but not Ctrl2, as was Ca^2+^-handling gene *CAMK2A*. In other Ca^2+^-handling genes, we observed increased *RYR2* expression and reduced *CASQ2* and *SLN* expression in *DSG2*^Mut^ CMs compared to controls.

Immunofluorescent imaging illustrated reduced overall signal intensity of desmoglein-2, but also revealed reduced localization at the cell membrane in conjunction with perinuclear accumulation in *DSG2*^Mut^ hiPSC-CMs (Figure 3B). The latter finding suggests defects in trafficking of desmoglein-2 in these cells, as previously reported in human and animal models of ARVC [40,41]. Western immunoblot analysis demonstrated much lower expression of full-length (~130kDa) desmoglein-2 in *DSG2*^Mut^ CMs, but retained lower molecular weight bands (Figure 3F,G). 

We found mRNA abundance of desmosomal components *PKP2* and *JUP* to be slightly increased and unaffected, respectively, in *DSG2*^Mut^ cells, but observed no difference in protein localization of either marker by immunostaining (Figure 3C,D) or expression by Western blot studies (Figure 3F,G). We also measured the abundance of two key calcium handling proteins, CaM Kinase II and PKA-C in their active (i.e. phosphorylated) forms (pCaMKII and pPKA-C) and found pCaMKII abundance was not significantly different among the three lines. *DSG2*^Mut^ hiPSC-CMs had levels of pPKA-C similar to those in Ctrl1, but greater than those in Ctrl2 (Figure S3). Immunostaining for α-actinin and cardiac troponin I revealed that *DSG2*^Mut^ hiPSC-CMs displayed narrower myofibrils compared to control hiPSC-CMs (Figure 3E), although Western blots demonstrated that α-actinin protein abundance was not altered in *DSG2*^Mut^ CMs. To assess myofibril diameter, we measured the length of Z-lines, denoted by α-actinin immunopositivity, in randomly selected regions across several 100X confocal images. We found myofibril diameter in both control lines to be in agreement with measurements reported in the literature [42] but confirmed that Z-line length, i.e. myofibril width, was significantly shorter in *DSG2*^Mut^ CMs compared to controls (0.76 ± 0.08 µm in *DSG2*^Mut^ vs. 1.17 ± 0.2 µm in Ctrl1 and 1.22 ± 0.23 µm in Ctrl2; Figure 3H). 

### 3.4. Cytokine and Chemokine Expression in DSG2^Mut^ hiPSC-CMs

**Figure 4:**
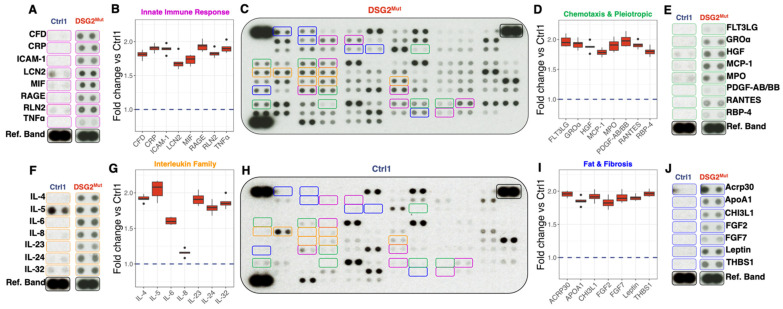
Cytokine expression in *DSG2*^Mut^ hiPSC-CMs. (**A**,**C**,**E**,**F**,**H**,**J**) Representative cytokine immunoblots from wildtype control (Ctrl1) and *DSG2*^Mut^ hiPSC-CMs, grouped based on their role in inflammation: (**A**) the innate immune response (purple); (**E**) chemoattractant and pleiotropic regulation (green); (**F**) interleukins that regulate immune cell differentiation and proliferation (orange); and (**J**) adipo-fibrokines involved in lipid accumulation and metabolism and fibrotic remodeling (blue). (**B,D,G,I**) Box and whisker plots of selected cytokines from Figure 4A,E,F,J. Data presented as mean ± standard deviation.; Blue dashed line represents normalized values for Ctrl1; Ctrl1 (*n* = 4); *DSG2*^Mut^ (*n* = 6); For all values shown in (**B**,**D**,**G**,**I**) *p* < 0.05 comparing *DSG2*^Mut^ vs Ctrl1.

Myocardial inflammation is a recurrent pathological hallmark of ARVC, even presenting as myocarditis before subsequent genetic screening confirms a diagnosis of ARVC [43]. Considering we previously showed that PKP2-mutant hiPSC-CMs produce and secrete potent inflammatory cytokines via nuclear factor kappa-B (NFĸB)-mediated transcription [15], we assessed both hiPSC-CM lysates and cell culture supernatants for the production and secretion, respectively, of more than 100 cytokines in *DSG2*^Mut^ and control cell lines. We identified eight cytokines in *DSG2*^Mut^ hiPSC-CMs that are involved in the innate immune response and complement component system that were upregulated in *DSG2*^Mut^ hiPSC-CM lysates (Figure 4A-C,H). Included among these was the receptor for advanced glycation end products (RAGE), which binds to nuclear high-mobility group box-1 (HMGB1) to promote cell death [44]; this is consistent with our recent work demonstrating that HMGB1-nuclear loss leads to myocyte cell necroptosis in *Dsg2*-mutant mice [14].

We identified numerous upregulated pleiotropic cytokines and chemokines involved in mobilizing and recruiting immune cells (Figure 4D,E), and members of the potent, proinflammatory interleukin (IL)-family (Figure 4F,G). Of these, monocyte chemoattract protein-1 (MCP-1) was upregulated in *DSG2*^Mut^ hiPSC-CMs, a finding previously reported in *Dsg2*-mutant mouse myocardium and in hiPSC-CMs and cell culture supernatant from PKP2-mutant hiPSC-CMs [15]. Of the members of the IL-family, cytokines that regulate immune cell differentiation (e.g., IL-5, IL-6, and IL-32) and proliferation (e.g., IL-4, IL-23, IL-24) were elevated in *DSG2*^Mut^ CMs (Figure 4F,G). Lastly, several adipo-fibrokines that drive lipid accumulation, regulate adipocyte survival and proliferation, and promote fibrotic remodeling were significantly elevated in *DSG2*^Mut^ hiPSC-CMs compared to controls (Figure 4I,J); however, Oil Red O staining did not reveal lipid deposition in mutant or control hiPSC-CMs (Figure S4).

### 3.5. Immune Signaling in DSG2^Mut^ hiPSC-CMs Is Regulated by Canonical and Non-Canonical NFĸB Pathways

**Figure 5:**
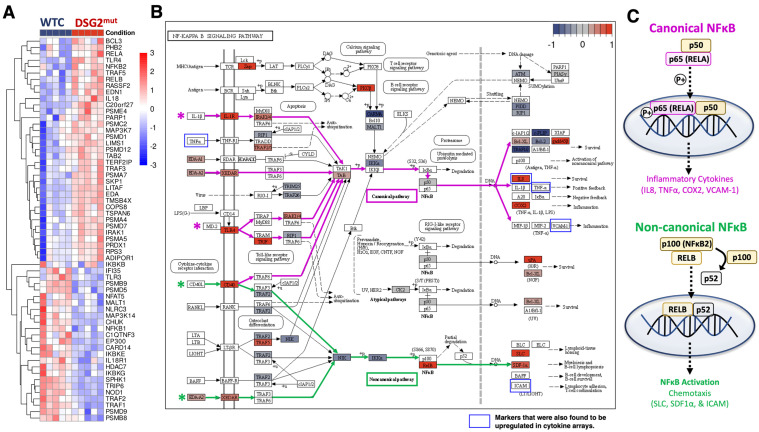
Bulk RNA-Seq analysis reveals both canonical and non-canonical NFĸB pathways regulate immune signaling in *DSG2*^Mut^ hiPSC-CMs. (**A**) RNA-Seq heatmap analysis and (**B**) KEGG NFĸB pathway schematic. For (**A**), we identified genes in the NFĸB pathway that were differentially expressed between Ctrl1 and *DSG2*^Mut^ hiPSC-CMs (BH-adjusted *p*-value < 0.05). NFĸB pathway genes were identified using genes associated with the gene ontology term “NIK/NF-kappaB signaling” (GO:0038061). Color scale denotes row-scaled log expression values. For (**B**), purple lines and arrows indicate canonical NFĸB pathway; green lines and arrows indicate non-canonical NFĸB pathway; red shading indicates higher expression in *DSG2*^Mut^ relative to control, blue indicates lower expression, and gray indicates no difference. (**C**) Schematic demonstrating canonical NFĸB signaling through RELA and non-canonical NFĸB signaling through RELB.

Our prior studies demonstrated that both myocardial inflammation in *Dsg2*-mutant mice and cytokine production in PKP2-mutant hiPSC-CMs are regulated via the canonical NFĸB pathway [15]. We performed bulk RNA-Seq (Figure 5A) from normal and *DSG2*^Mut^ hiPSC-CMs and applied KEGG PATHWAY analysis to map out the components of the NFĸB pathway (Figure 5B). Our results implicate four cell surface receptors involved in canonical and non-canonical NFĸB signaling that were upregulated in *DSG2*^Mut^ CMs: IL1R, XEDAR, Toll-like receptor-4 (TLR4), and CD40 (Figure 5A,B). TLR4 is a pleiotropic NFĸB transcription factor regulating IL-23-induced T-cell infiltration [45], a cytokine we also found upregulated in *DSG2*^Mut^ hiPSC-CMs (Figure 4F,G). Downstream of TLR4- and IL1R-induced NFĸB activation, we found IL1R kinase-1 (IRAK1) and TRIF to also be upregulated in *DSG2*^Mut^ CMs (Figure 5A,B). These two pathways merge to signal through TGFβ activating kinase (TAK1/MAP3K7) and TAK binding protein (TAB) to activate p50/p65 (RELA) canonical NFĸB (Figure 5A–C). While RELA itself was not differentially expressed via RNA-Seq analysis, RELA-mediated transcripts (e.g., IL8, COX4, Figure 5A,B) were upregulated in *DSG2*^Mut^ hiPSC-CMs. This discrepancy is potentially explained by regulatory differences imposed by post-transcriptional mechanisms. 

We next focused our attention to the non-canonical cell surface receptors, CD40 and XEDAR. Non-canonical NFĸB signaling is mediated through tumor necrosis factor receptor-associated factors (TRAF1–6), where prior work demonstrated TRAF2 acts as a negative regulator of NFκB [46,47,48]. Our data showing reduced levels of TRAF2 (Figure 5A,B), and upregulation of RELB, a positive regulator of non-canonical NFĸB signaling (Figure 5A,B), is consistent with the role of non-canonical NFĸB signaling in this model of ARVC. Furthermore, while not significantly differentially expressed, NFĸB1 (p105) and TRIP6 (NFĸB suppressors) were both downregulated in *DSG2*^Mut^ hiPSC-CMs (Figure 5A,B).

### 3.6. Shortened Action Potential Durations, Heterogeneous Excitation, and Altered Calcium Handling in DSG2^Mut^ CMs

**Figure 6:**
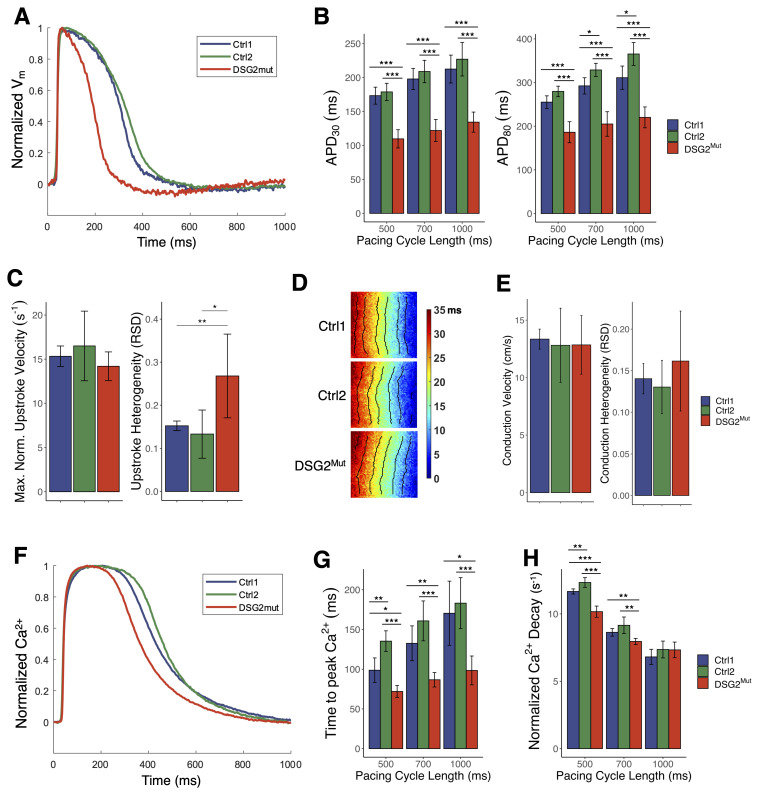
Action potential characteristics of hiPSC-CM monolayers measured via optical mapping. (**A**) Representative traces of normalized transmembrane potential (V_m_). (**B**) Action Potential Duration (APD) measured at 30% and 80% repolarization. (**C**) Relative upstroke velocity measured at 1000ms pacing and spatial heterogeneity of upstroke velocity, calculated as the relative standard deviation (RSD) of all measurements within each sample. (**D**) Activation maps with isochrone lines spaced 5ms apart. (**E**) Mean conduction velocity and conduction heterogeneity measurements at 1000 ms pacing. (**F**) Representative normalized calcium traces. (**G**) Time-to-peak Ca^2+^. (**H**) Ca^2+^ decay rate, measured using normalized data. V_m_ mapping (**B**–**E**): Ctrl1 (*n* = 8), Ctrl2 (*n* = 13) *DSG2*^Mut^ (*n* = 9). Ca^2+^ mapping (**G,H**): Ctrl1 (*n* = 10), Ctrl2 (*n* = 7), *DSG2*^Mut^ (*n* = 9). * *p* < 0.05; ** *p* < 0.01, *** *p* < 0.001.

On day 28 of differentiation, hiPSC-CMs from each line were plated onto coverslips and incubated for 9–11 days to allow a confluent monolayer to develop. At day 37–39, optical mapping recordings were taken under electrical pacing to measure the action potential and calcium transient characteristics of the monolayers. Action potential duration at 80% repolarization (APD_80_) was significantly reduced in *DSG2*^Mut^ CMs compared with both control lines (220 ±24 ms in *DSG2*^Mut^ vs 311 ± 27 ms in Ctrl1 and 365 ± 26 ms in Ctrl2 at 1000ms pacing) as was APD_30_ (134 ± 15 ms in *DSG2*^Mut^ vs 213 ± 21 ms in Ctrl1 and 227 ± 25 ms in Ctrl2 at 1000 ms pacing; Figure 6A,B), revealing important differences in repolarization. A possible basis for the APD differences is suggested by RT-qPCR analysis, which showed that mRNA expression of *KCNQ1* was higher in the *DSG2*^Mut^ CMs compared to Ctrl1 (Figure 3A). *KCNQ1* encodes the ion channel that mediates *I_Ks_*, a key repolarizing current in cardiomyocytes. 

Optical mapping experiments showed similar conduction velocities and conduction heterogeneity in all lines (Figure 6D,E) with no significant differences in relative upstroke velocity (Figure 6C). However, we did find that the upstroke velocity in the *DSG2*^Mut^ hiPSC-CMs was more variable across the monolayer within each sample (0.27 ± 0.1 relative standard deviation (RSD) in *DSG2*^Mut^ vs 0.15 ± 0.01 in Ctrl1 and 0.13 ± 0.06 in Ctrl2 at 1000 ms pacing; Figure 6C). This suggests substantial spatial heterogeneity of excitability or electrical coupling in these cells, though the latter seems less likely because of the lack of measurable differences in conduction heterogeneity (Figure 6E). 

In addition, mapping the relative intracellular calcium concentration in hiPSC-CM monolayers during excitation revealed significant differences in calcium dynamics. Time to peak Ca^2+^ was considerably shorter in *DSG2*^Mut^ CMs than in controls (98 ± 18 ms in *DSG2*^Mut^ vs 170 ± 40 ms in Ctrl1 and 183 ± 32 ms in Ctrl2; Figure 6G). At faster pacing rates *DSG2*^Mut^ CMs also displayed slowed Ca^2+^ transient decay compared to controls (Figure 6H), though this effect was not observed at 1000ms pacing. Notably, RT-qPCR analysis demonstrated significant differences in expression of several calcium handling genes in *DSG2*^Mut^ hiPSC-CMs -- namely, increased mRNA expression of ryanodine receptor 2 (*RYR2*) along with lower expression of sarcolipin (*SLN*) and calsequestrin (*CASQ2*) (Figure 3A).

### 3.7. DSG2 Knockdown Recapitulates Some, but Not All, Effects of DSG2 c.2358 Variant in Control hiPSC-CMs

**Figure 7:**
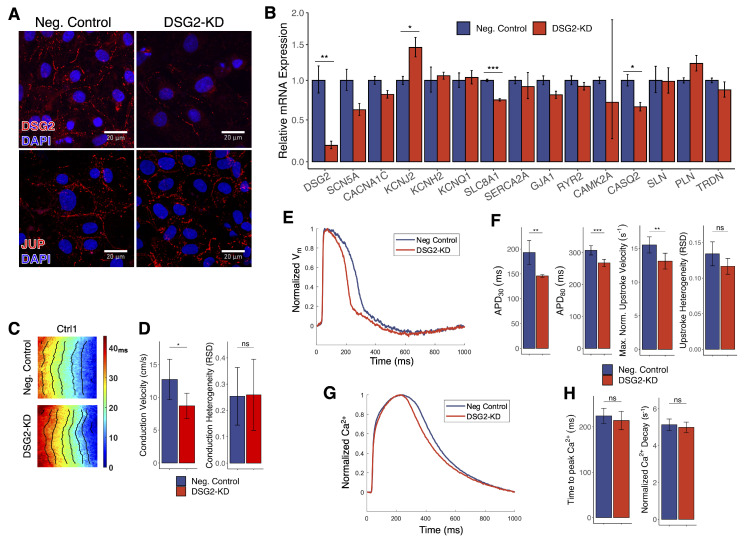
Effects of *DSG2* suppression on Ctrl1 hiPSC-CMs. (**A**) Immunostaining for desmoglein-2 and plakoglobin in Ctrl1 hiPSC-CMs treated with anti-*DSG2* and negative control siRNA. White scale bars, 20 µm. (**B**) RT-qPCR measurements of key transcripts in Ctrl1 hiPSC-CMs with and without *DSG2* knockdown (**C**) Activation maps with 5 ms isochrones and (**D**) mean conduction velocities and conduction heterogeneity of Ctrl1 hiPSC-CMs with *DSG2* knockdown (**E**) Representative V_m_ traces of Ctrl1 hiPSC-CMs with *DSG2* knockdown and (**F**) mean action potential characteristics. (**G**) Representative Ca^2+^ traces for Ctrl1 hiPSC-CMs with *DSG2* knockdown and (**H**) mean Ca^2+^ transient characteristics. Neg. Control (*n* = 9); DSG2-KD (*n* = 8). * *p* < 0.05, ** *p* < 0.01, *** *p* < 0.001, ns = no significant difference.

To interrogate the role that overall *DSG2* expression plays in mediating the biomolecular and electrophysiological changes observed in the *DSG2*^Mut^ hiPSC-CMs, we used small interfering RNA (siRNA) to suppress expression of *DSG2* in control cells. After 72 h, siRNA transfection of Ctrl1 hiPSC-CMs substantially reduced *DSG2* expression at both the transcriptional and protein levels, with an 81% decrease in mRNA abundance (Figure 7A,B) which is a greater reduction than presents in the *DSG2*^Mut^ cells at baseline relative to controls. We did not observe a significant change in the localization of desmoglein-2 in response to *DSG2* knockdown (DSG2-KD), nor did we find any effect on the expression or localization of plakoglobin (Figure 7A). 

*DSG2* knockdown resulted in altered abundance of RNAs encoding several ion channels including reduced *SLC8A1* (I_NCX_) and increased *KCNJ2* (I_K1_) (Figure 7B), but notably its effects on *SCN5A* and *KCNQ1* transcript levels, which were altered in *DSG2*^Mut^ CMs (Figure 3A), were not statistically significant. Expression of calcium handling genes *RYR2*, and *SLN*, which were all differentially expressed in *DSG2*^Mut^ CMs, were not significantly affected by *DSG2* suppression.

Optical mapping showed that *DSG2* knockdown in healthy hiPSC-CM monolayers shortened the action potential duration (Figure 7E,F). At 1000 ms pacing rates, APD_30_ was reduced by 47 ms and APD_80_ was reduced by 40 ms in Ctrl1 CMs, similar to the differences seen between untreated control and *DSG2*^Mut^ CMs. However, unlike our observation in *DSG2*^Mut^ cells, *DSG2*-suppressed Ctrl1 cells displayed slower conduction velocities (Figure 7C,D) and slower AP upstroke velocity, without significant effect on the spatial heterogeneity of either parameter (Figure 7F). Interestingly, similar action potential and conduction effects were observed in *DSG2*^Mut^ hiPSCs in response to *DSG2* knockdown (Figure S7). We also found that calcium transients in Crtl1 CMs were not substantively altered by *DSG2* knockdown; times-to-peak Ca^2+^ and Ca^2+^ decay rates were similar between treated and untreated monolayers (Figure 7G,H).

## 4. Discussion

In this study we established a novel patient-specific hiPSC model of ARVC from an individual with a pathogenic variant in *DSG2*, the second most common gene associated with ARVC [49]. We performed a broad characterization of cardiomyocytes derived from these hiPSCs, evaluating their biomolecular features as well as their electrophysiological behaviors in monolayer cultures. Our studies confirmed reduced *DSG2* expression and disrupted protein localization in *DSG2*^Mut^ CMs, with no apparent changes in expression or localization of other key desmosomal components. We also found that these cells have aberrant electrophysiology and altered Ca^2+^ handling when compared to wild-type cells, including shortened action potential duration and time-to-peak calcium, as well as altered immune cytokine expression.

The *DSG2* c.2358delA variant results in a frameshift and early termination in the intracellular cadherin-like domain of DSG2. It was previously demonstrated that the more distal C-terminal domains are critical in stabilizing desmoglein-2 at the cell membrane, and that deletion of those downstream regions results in increased protein internalization and consequently weakened cell-cell cohesion [50]. Our observations are consistent with those findings: we found desmoglein-2 accumulates intracellularly (primarily perinuclear) in CMs from the mutant line, whereas it was predominantly localized at myocyte-myocyte junctions in controls.

Late-stage ARVC has several well-established pathological markers in human myocardium; however, establishing a definition and criteria constituting a faithful in vitro model of ARVC has proven difficult. Some authors have proposed using reduced plakoglobin expression at cell boundaries as a key component of that definition, since this feature has been shown in both animal [40] and hiPSC [51] models and has even been evaluated as a potential diagnostic marker in humans [52,53,54]. However, plakoglobin dysregulation has not been observed universally, and notably the extent of dysregulation has been variable among pathogenic variants studied. In our model, plakoglobin expression was not appreciably altered in transcription, protein abundance, or in its localization. Interestingly though, myofibril organization did seem to be affected in *DSG2*^Mut^ hiPSC-CMs, as we found that myofibrillar diameter was significantly reduced in these cells compared to control lines. Studies of myocardial biopsies of ARVC patients have shown altered or degraded myofibrillar structure and some forms of ARVC are associated with myofibrillar myopathy [55,56]. If similar myofibrillar differences are found in other hiPSC models of ARVC, it could offer a novel criterion for defining ARVC phenotype in vitro.

Inflammation is a pathological hallmark of ARVC and occurs during so called “hot phases” of ARVC pathogenesis, further contributing to cardiac remodeling, myocyte cell death and myocardial fibrotic replacement [57]. It is not yet known whether these inflammatory periods are the result of extrinsic factors (e.g., immune cell infiltration) or intrinsic CM dysregulation (e.g., NFĸB-mediated cytokine secretion from CMs), but they likely involve both. Specifically, prior reports show infiltration of immune cells in hearts of animal models of ARVC [14,15] and patients with ARVC [58], indicating that extrinsic inflammatory pathways are activated. Yet, our cytokine data in *DSG2*^Mut^ hiPSC-CMs largely corroborates prior reports in PKP2-mutant hiPSC-CMs [15] and clearly demonstrate an intrinsic, NFĸB-mediated inflammatory pathway that is dysregulated in hiPSC-CMs, independent of nonmyocyte infiltration. Considering the numerous chemotactic cytokines and inflammation-associated gene transcripts upregulated in purified *DSG2*^Mut^ hiPSC-CM cultures when compared to wild-type cells, this intrinsic pathway may be the driving force for immune cell recruitment to ARVC hearts. Specifically, our RNA-Seq data supports a causative role for canonical NFκB signaling through an IRAK-TAB/TAK-RELA pathway and non-canonical NFκB signaling through a TRAF-IKKa (CHUK) -RELB pathway [14]. More broadly, the cytokine profiles in these *DSG2*^Mut^ cells indicates a global upregulation of inflammation machinery and immune cell recruitment. For example, LIF—also known as differentiation stimulating-factor (DSF)—was found to be upregulated in *DSG2*^Mut^ hiPSC-CMs and is a strong inducer of hematopoietic cell differentiation, e.g., myeloid cells into macrophages or pleiotropic cells into T-cells. Both of these terminally differentiated immune cells are routinely found in ARVC hearts [58]. We also found myeloperoxidase and growth regulated oncogene alpha (GROα) to be upregulated in *DSG2*^Mut^ hiPSC-CMs; both of these cytokines are powerful contributors to cardiac fibrosis via recruitment of neutrophils (immune signaling) together with direct effects on fibroblast proliferation and differentiation [56]. 

Prior studies of ARVC hiPSC-CMs have reported a range of electrophysiological characteristics. An hiPSC-CM model harboring a compound pathogenic variant in *PKP2* showed abnormal sodium channel function as indicated by slowed upstroke speeds and reduced total sodium current [24]. A study of DSG2 p.G638R hiPSC-CMs found similar defects, and another study evaluating hiPSC-CMs derived from a patient with a rare homozygous DSG2 mutation (p.R119X) found very slow conduction velocities compared to an isogenic line with one wildtype *DSG2* allele [25,59]. In our model, we did not observe any significant differences between the *DSG2*-mutant CMs and control CMs in terms of their conduction velocity or relative AP upstroke velocity, though we did find that the *DSG2*^Mut^ CMs displayed much greater upstroke heterogeneity within each monolayer compared to the controls. Because the cardiomyocyte monolayers used in this study possess electrically connected myocytes and syncytial behavior, our metrics of excitability also reflect source-load current effects and therefore can differ from single-cell measurements of excitability. Nevertheless, when considered alongside our finding that expression of the cardiac sodium channel (*SCN5A*) is not reduced in *DSG2*^Mut^ cells, these results are inconsistent with the findings of major intrinsic sodium current dysfunction reported in those prior studies.

In the study of DSG2 p.G638R hiPSCs, the authors also found reduced expression and activity of multiple potassium channels, including *I*_to_, *I*_SK_, and *I*_KATP_ [25]. Whereas they did not report any significant difference in repolarization dynamics in those cells, we measured substantially shortened action potential durations in our *DSG2*-mutant CMs as compared to controls. *DSG2*^Mut^ CMs displayed slightly higher mRNA expression of *KCNQ1*, the gene encoding the channel responsible for *I*_Ks_, which is one of the major repolarizing currents of the action potential. *DSG2*^Mut^ CMs also had significantly shorter time-to-peak calcium, which may be a consequence of the shortened action potential duration. Interestingly, the ECG of the donor of our *DSG2*^Mut^ line fulfilled the “major” criteria for ARVC-related repolarization abnormality due to T wave inversions (Figure 1B, Table 1), but had no depolarization abnormality. Given that altered electrophysiology at the cellular level may be reflected in patient ECGs [60], our findings of shortened repolarization times but unchanged conduction velocity and upstroke times are consistent with the ECG findings.

One possible explanation for the differences in action potential characteristics in our model compared to other *DSG2*-mutant models may be related to alterations in ion channel trafficking, which may be linked to desmoglein-2 and plakophilin-2 trafficking through the formation of macromolecular complexes, such as between Nav1.5 and plakophilin-2 [61], Nav1.5 and desmoglein-2 [11], and Kir2.1 and filamin [62]. The different action potential behavior in our model as compared to other *DSG2*-mutant models, could be explained by variation in how different mutations affect the ability of DSG2 to form these complexes.

Abnormal Ca^2+^-handling can promote arrhythmia via a number of different mechanisms and has been implicated in acquired and inherited cardiovascular diseases [63]. Previously reported hiPSC-CM models of ARVC have exhibited accelerated calcium relaxation [23], disrupted calcium homeostasis [17], and increased susceptibility to epinephrine-induced proarrhythmic events (i.e., delayed afterdepolarizations) [25]. Our findings further support the theory that cardiomyocyte calcium regulation is altered in ARVC. In addition to displaying much shorter time-to-peak Ca^2+^ and slower Ca^2+^ decay, RT-qPCR experiments showed overexpression of *RYR2* and reduced expression of *SLN*, which encode the ryanodine receptor and sarcolipin, respectively. Ryanodine receptors regulate calcium release from the sarcoplasmic reticulum (SR), and sarcolipin regulates SR calcium uptake; both are critical for calcium homeostasis [64,65,66]. High levels of cytosolic Ca^2+^ have been shown to enhance *I*_Ks_ in rabbit cardiomyocytes, which could possibly explain the altered expression of *KCNQ1*, as well as the shortening of action potential duration observed in *DSG2*^Mut^ hiPSC-CMs [67].

In our model, overall expression of *DSG2* was lower in the *DSG2*^Mut^ CMs than in the controls. To isolate the contributions of a gross difference in *DSG2* expression from the potentially dominant negative effects of the c.2358delA variant, we used siRNA targeted against *DSG2* mRNA to suppress *DSG2* expression without introducing the potential for truncated protein translation. Global suppression of *DSG2* in Ctrl1 CMs resulted in shortened action potential durations but had little effect on Ca^2+^ dynamics. These AP changes are fairly similar to the behavior seen in *DSG2*^Mut^ hiPSC-CMs, but mRNA expression patterns suggest that the AP shortening in the two cases could be the result of different mechanisms. While *DSG2*^Mut^ hiPSC-CMs expressed more of the gene encoding I_Ks_ compared to control, *DSG2* knockdown did not alter expression in that gene but instead resulted in increased expression of KCNJ2, which encodes I_K1_. RT-qPCR data also offers an explanation for the minimal effects of *DSG2* knockdown on calcium dynamics in these cells. Although expression of CASQ2 was reduced by *DSG2* knockdown, the other calcium handling genes that were differentially regulated in *DSG2*^Mut^ cells – RYR2 and SLN – were not affected. 

While we observed no conduction differences between *DSG2*^Mut^ and control hiPSC-CMs at baseline, we found that *DSG2* knockdown caused conduction slowing in control lines. Reduced expression of *DSG2* can disrupt connexin-43 localization; indeed, one study showed that mice with a cardiospecific knockout of *Dsg2* developed prolonged PR and QRS intervals on ECG recordings, indicative of significant conduction delay [68]. Interestingly, *DSG2* knockdown caused similar levels of conduction slowing control and *DSG2*^Mut^ hiPSC-CMs (Figure S7). These results suggest that direct suppression of *DSG2* expression is more disruptive of conduction than the *DSG2* c.2358delA variant alone.

### Study Limitations

hiPSC-CMs, while a valuable tool, demonstrate a relatively immature phenotype, differing from adult cardiomyocytes phenotypically in several ways. In typical monolayer cultures, they do not assume an elongated morphology or form fully organized intercalated discs, which could affect the level and spatial distribution of desmosomal protein expression. Furthermore, these cells beat spontaneously, and because of their immaturity are more depolarized at rest than adult ventricular cells, which can affect the dynamics of voltage-gated ion channels and alter excitability [69]. On the other hand, hiPSC-CMs cultured in a syncytium such as ours may possess resting transmembrane potentials more similar to adult cardiomyocytes, as compared to single cell preparations [70,71]. We also note that our RT-qPCR studies are limited to three biological replicates for each group. Another limitation is that hiPSC-CMs from the ARVC donor were compared to those from unrelated normal donors, so differences in genetic background are potentially confounding. However, this risk is ameliorated by the use of two independent control lines. Finally, our electrophysiological characterization was limited by the fact the optical mapping system used provided relative, but not absolute, measures of transmembrane potential and Ca^2+^ concentration.

## 5. Conclusions

In this study we have described an hiPSC-CM model of ARVC derived from a patient with a novel pathogenic mutation in *DSG2* and demonstrated a distinct phenotype with altered *DSG2* expression, enhanced inflammatory signaling, and electrophysiological aberrations compared with controls. The *in vitro* repolarization and conduction features correlated with the donor’s clinical features, and direct suppression of *DSG2* in control CMs reproduced many of the mutant line’s most prominent phenotypic features. Our findings are generally consistent with observations of ARVC in human studies but differ in several aspects from other published hiPSC-CM models of the disease. This work contributes new evidence that in vitro models of ARVC vary in their behavior significantly, just as ARVC is known to present variably in patients, and underscores the challenge of defining ARVC in vitro in a way that is generalizable across models. Patient-specific hiPSC studies offer the opportunity to systematically investigate this phenotypic variation, which could allow investigators to identify pathophysiological pathways that are common across the disease and to develop broadly useful therapies. Such studies could also provide important insight into attributes of ARVC and other forms of ACM that are specific to different pathogenic variants, aiding the design of more narrowly targeted treatments.

## Figures and Tables

**Table 1:** Demographic data and clinical history of patient JHU013. 2010 Task Force Criteria were used.

Attribute	Finding
Demographic	
Gender	Female
Race/Ethnicity	South Asian
Presentation (age [yrs]: symptoms)	22: anterior precordial T wave inversions noted on ECG. 32: dizziness, palpations
Age at diagnosis (yrs)	45
Age at last follow-up (yrs)	51
2010 Task Force Criteria Fulfillment [26]	
ECG Repolarization Abnm.	Major: Inverted T waves in right precordial leads (V_1_, V_2_, and V_3_)
ECG Depolarization Abnm.	None
Arrhythmia	Minor: >500 PVCs per 24 h on Holter monitor
Structural	Major: Regional RV dyskinesia + RV EDVi > 100 ml/m^2^
Family History	Major: ARVC confirmed in a first-degree relative who meets current Task Force criteria
Cardiac MRI (at last follow-up)	
RV EDVi (ml/m^2^)	118
RV EDVi / LV EDVi	2.62
RV wall-motion Abnm.	Regional dyskinesia, aneurysms
LV wall-motion Abnm.	None
RVEF (%)	44
LVEF (%)	57
Delayed enhancement	Extensive delayed enhancement in RV free wall and inferior wall
Events	
Sustained ventricular arrythmia	No
Cardiac transplant	No
Death	No

Abnm.: abnormalities, RV: right ventricle, LV: left ventricle, RVEF: right ventricular ejection fraction, LVEF: left ventricular ejection fraction, EDVi: end diastolic volume indexed to body surface area.

## Data Availability

All raw sequencing data as well as count tables and sample information are available on GEO at accession number: GSE176209. Code to replicate results from this manuscript can be found at https://github.com/skannan4/dsg2-mut.

## Supplementary Materials

The following are available online at https://www.mdpi.com/article/10.3390/jcm10143061/s1, Figure S1: Cardiac MRI for patient JHU013, Figure S2: Cardiac Troponin T expression in hiPSC-CMs, Figure S3: hiPSC-CM expression of pPKA-C and pCaMKII, Figure S4: Lipid production in hiPSC-CMs, Figure S5: Cytokine heatmaps from hiPSC-CM lysates and cell culture supernatants, Figure S6: Conduction velocity aspect ratio in hiPSC-CM monolayers, Figure S7: Optical mapping analysis of siRNA-mediated *DSG2* knockdown in *DSG2*^Mut^ hiPSC-CMs, Table S1: RT-qPCR primer sequences, Table S2: siRNA oligo sequences, Table S3: Primary antibodies for immunocytochemistry and Western blot, and Supplementary Methods.
