# Supplementary material for: Altered Electrical, Biomolecular, and Immunologic Phenotypes in a Novel Patient-Derived Stem Cell Model of Desmoglein-2 Mutant ARVC
Source: J Clin Med. 2021 Jul 10;10(14):3061. doi: 10.3390/jcm10143061 (PMC8306340; doi:10.3390/jcm10143061)
Supplement: Supplementary file 1 [file jcm-10-03061-s001.zip › jcm-1259329-supplementary.pdf]

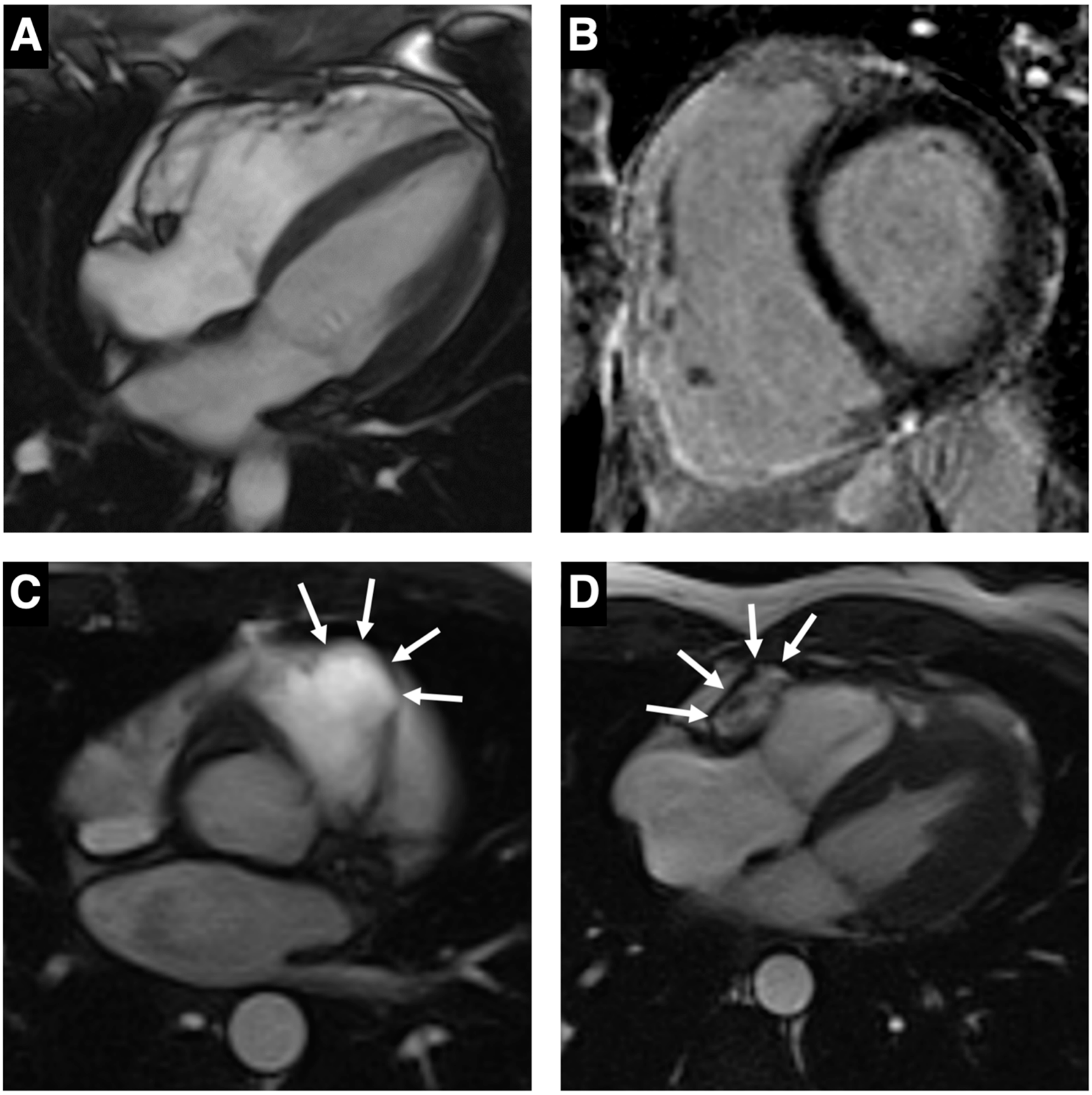


**Figure S1. Cardiac MRI for patient JHU013.** Images obtained at last clinical follow-up. **(A)** Four-chamber bright blood steady-state free precession cardiac MRI (CMR) image showing mild RV enlargement. **(B)** Extensive RV enhancement of the anterior and inferior walls on short axis post-contrast late enhancement CMR. **(C-D)** End-systolic axial images obtained from cine CMR showing regions of RV outpouching secondary to dyskinesia (arrows).

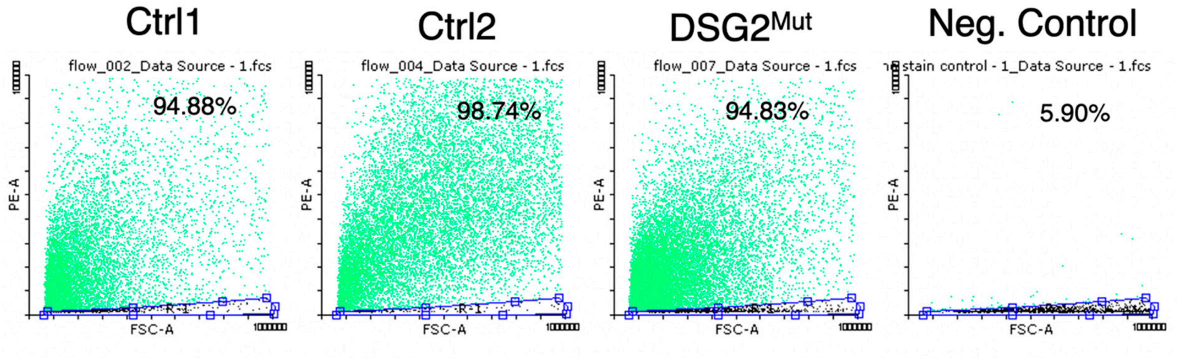


**Figure S2. Cardiac Troponin T expression in hiPSC-CMs.** iPSC-CMs were fixed on differentiation day 35, stained for cardiac troponin T and analyzed via flow cytometry. CMs from all 3 lines demonstrated >90% cTnT-positivity.

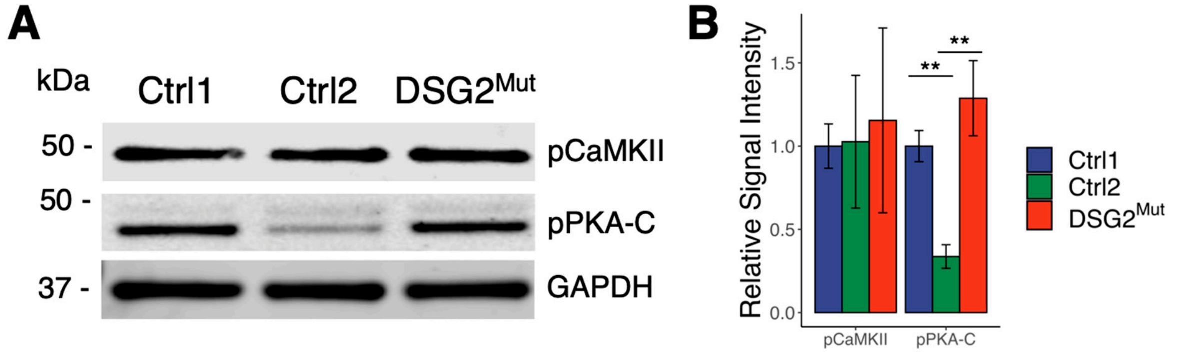


**Figure S3. hiPSC-CM expression of pPKA-C and pCaMKII. (A)** Representative images of Western blots from all three hiPSC-CM lines, probed for the active (i.e. phosphorylated) forms of PKA-C and CaMKII. **(B)** Quantification of western blot signal intensity relative to GAPDH and normalized to Ctrl1. n=3 for each line. **p<0.01.

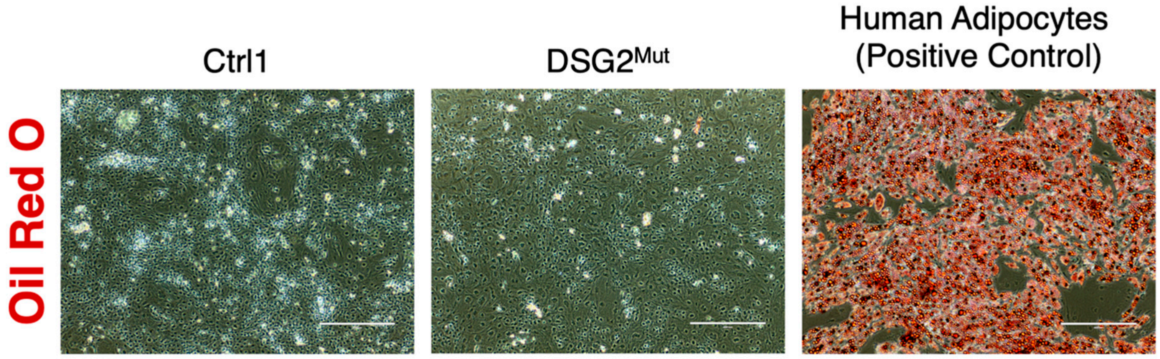


**Figure S4. Lipid production in hiPSC-CMs**. Ctrl1 and *DSG2*$^{Mut}$ hiPSC-CMs were fixed on differentiation day 35 and stained with Oil Red O (MilliporeSigma, St. Louis, MO, USA) to highlight lipid deposits. Phase contrast images revealed no positive staining in hiPSC-CMs from either line. Primary human adipocytes are presented as a positive control. White scale bar, 400 µm.

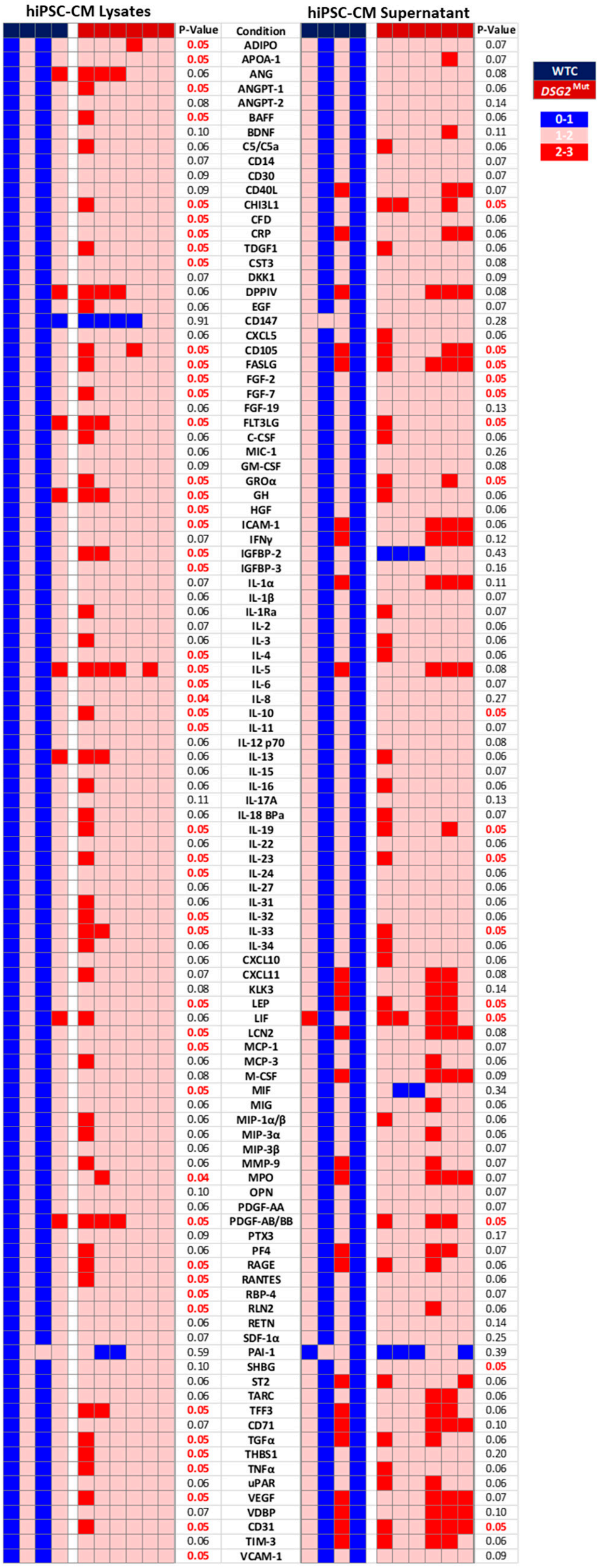


**Figure S5: Cytokine heatmaps from hiPSC-CM lysates and cell culture supernatants.** Heatmaps of 111 cytokines from wild-type control (Ctrl1) and *DSG2*$^{Mut}$ hiPSC-CM lysates (**Left Panels**) and cell culture supernatants (**Right Panels**) measured via printed antibody membrane arrays. Data presented as heatmaps normalized to Ctrl1. Fold change: <1.0 [blue]; 1.0-2.0 [pink]; and >2.0 [red]. Ctrl1 (n=4; dark blue header); *DSG2*$^{Mut}$ (n=6; dark red header); $p \leq 0.05$ (indicated in bold red text) for *DSG2*$^{Mut}$ using unpaired, two-tailed t-test.

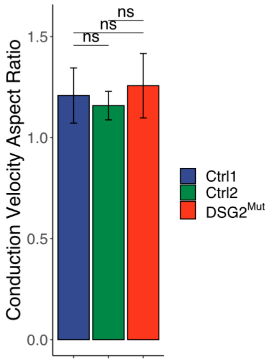


**Figure S6. Conduction velocity aspect ratio in hiPSC-CM monolayers.** Aspect ratio is determined by calculating the conduction velocity in the fastest direction and dividing it by the CV in the orthogonal direction. Values are not equal to zero as we'd expect in an isotropic population, because the line electrode used to stimulate the monolayers naturally produces faster conduction in the orthogonal direction than in the parallel direction due to increased liminal area. ns = no significant difference.

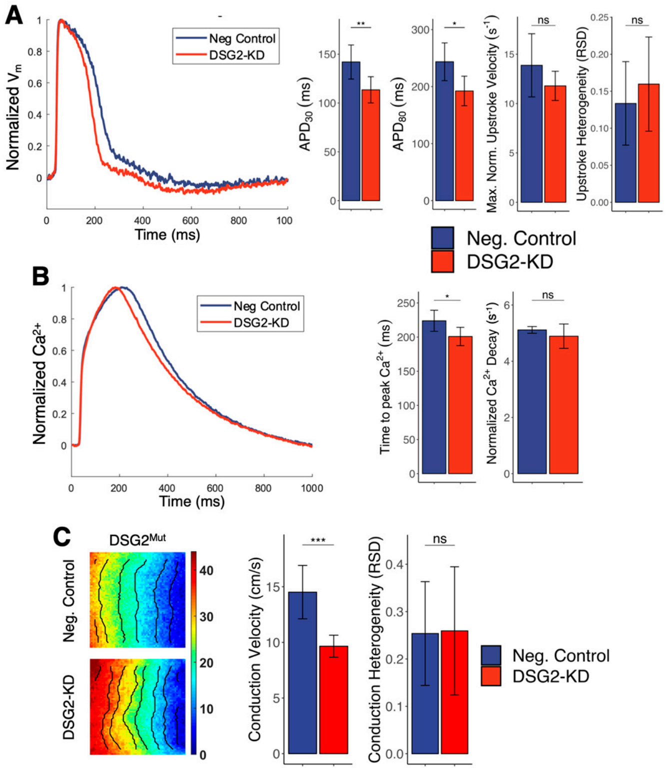


**Figure S7. Optical mapping analysis of siRNA-mediated *DSG2* knockdown in *DSG2*$^{Mut}$ hiPSC-CMs. (A)** Representative voltage traces of *DSG2*$^{Mut}$ hiPSC-CMs with and without *DSG2* knockdown and mean action potential characteristics. **(B)** Representative calcium traces and mean calcium transient characteristics. **(C)** Activation maps and mean conduction velocity of *DSG2*$^{Mut}$ hiPSC-CMs. Neg. Control (n=9); DSG2-KD (n=9). *$p < 0.05$, **$p < 0.01$, ***$p < 0.001$, ns = no significant difference.

**Table S1. RT-qPCR primer sequences**

| Gene | Forward | Reverse |
|---|---|---|
| **GAPDH** | 5’–GCACCGTCAAGGCTGAGAAC–3’ | 5’–TGGTGAAGACGCCAGTGGA–3’ |
| **18S rRNA** | 5’–CGGCTACCACATCCAAGGAA–3’ | 5’–GCTGGAATTACCGCGGCT–3’ |
| **ATP2A** | 5’–ATGGGGCTCCAACGAGTTAC–3’ | 5’–TTTCCTGCCATACACCCACAA–3’ |
| **CACNA1C** | 5’–GAAGCGGCAGCAATATGGGA–3’ | 5’–TTGGTGGCGTTGGAATCATCT–3’ |
| **CAMK2A** | 5’–ATTGAAGCCATAAGCAATGGAGA–3’ | 5’–TTCCGGGACCACAGGTTTTC–3’ |
| **CASQ2** | 5’–GGCAGAAGAGGGGCTTAATTT–3’ | 5’–GAAGACACCGGCTCATGGTAG–3’ |
| **DSG2** | 5’–ACGTAGAAGTTACGCGCATAAA–3’ | 5’–GGGTCACAATTCCTTCGTTAGTT–3’ |
| **GJA1** | 5’–ACAGGTCTGAGTGCCTGAAC–3’ | 5’–CGAAAGGCAGACTGCTCATC–3’ |
| **JCTN** | 5’–CATGGAGGACACAAGAATGGG–3’ | 5’–CCAAACGACAGCTACAGATGT–3’ |
| **JUP** | 5’–TCTCCAACCTGACATGCAACA–3’ | 5’–CATAGTTGAGACGCACAGAGTTC–3’ |
| **KCNH2** | 5’–TTCGACCTGCTCATCTTCGG–3’ | 5’–CGATGCGTGAGTCCATGTGT–3’ |
| **KCNJ2** | 5’–GTGCGAACCAACCGCTACA–3’ | 5’–CCAGCGAATGTCCACACAC–3’ |
| **KCNQ1** | 5’–GCGTCTCCATCTACAGCACG–3’ | 5’–GAAGTGGTAAACGAAGCATTTCC–3’ |
| **PKP2** | 5’–ATGACATGCTAAAGGCTGGCA–3’ | 5’–GGGAGCTGTACTGTGCTGTTC–3’ |
| **PLN** | 5’–ACCTCACTCGCTCAGCTATAA–3’ | 5’–CATCACGATGATACAGATCAGCA–3’ |
| **RYR2** | 5’–ACAACAGAAGCTATGCTTGGC–3’ | 5’–GAGGAGTGTTCGATGACCACC–3’ |
| **SCN5A** | 5’–GTGCCCAGAAGCAGGATGAG–3’ | 5’–GGACATACAAGGCGTTGGTG–3’ |
| **SLC8A1** | 5’–ACAACATGCGGCGATTAAGTC–3’ | 5’–GCTCTAGCAATTTTGTCCCCA–3’ |
| **SLN** | 5’–ATGGTCCTGGGATTGACTGAG–3’ | 5’–GTGCCCTCGGATGGAGAATG–3’ |
| **TRDN** | 5’–TCACAGAAGACATAGTGACGACG–3’ | 5’–TGGCAATAGAGCTTGCTGAAA–3’ |

**Table S2. siRNA oligo sequences.** Purchased from MilliporeSigma (St. Louis, MO, USA).

| Mat. # - Line | Forward | Reverse |
|---|---|---|
| **VC30002-001** | 5’-GAAAUUGAGCAGAGACAAA-3’ | 5’-UUUGUCUCUGCUCAAUUUC-3’ |
| **VC30002-004** | 5’-CUUCUACCUUGGUAGAUCA-3’ | 5’-UGAUCUACCAAGGUAGAAG-3’ |
| **VC30002-007** | 5’-CUACUCUGGCCAUGCCUAA-3’ | 5’-UUAGGCAUGGCCAGAGUAG-3’ |

**Table S3. Primary antibodies for immunocytochemistry and Western blot**

| Target | Manufacturer | Product Num. | Application |
|---|---|---|---|
| α-actinin (ACTN2) | Abcam | ab137346 | ICC, WB |
| Phsopho-CaMKII (The286) | Cell Signaling | | WB |
| Cardiac Troponin-I (cTnI) | United States Biological | T8665-13F | ICC |
| Desmoglein-2 (DSG2) | Proteintech | 21880-1-AP | ICC, WB |
| GAPDH | Abcam | ab9483 | WB |
| Nanog | BioLegend | 674001 | ICC |
| OCT3/4 | Santa Cruz Biotechnology | SC-5279 | ICC |
| Phospho-PKA C (Thr197) | Cell Signaling | 5661T | WB |
| Plakoglobin | Abcam | ab184919 | WB |
| Plakoglobin (JUP) | MiliporeSigma | P8087 | ICC |
| Plakophilin-2 (PKP2) | Abcam | ab220840 | ICC, WB |
| SOX2 | Thermo Fisher Scientific | 14-9811-80 | ICC |

ICC, Immunocytochemistry; WB, Western blot.

## Supplementary Methods

*Reprogramming PBMCs to generate hiPSC line*

Peripheral blood mononuclear cells (PBMCs) were isolated using Ficoll-Paque PLUS density gradient media (GE Healthcare, Waukesha, WI, USA) and plated at high density in PBMC growth media (PBMC-M; formulation listed below). PBMCs were expanded for 10 days before being harvested and reprogrammed using the Cytotune 2.0 Sendai virus kit containing Yamanaka factors (Thermo Fisher Scientific, Waltham, MA, USA), per manufacturer's protocol. After 24 hours, the cells were resuspended in PBMC-M media and seeded into Synthemax-treated tissue plates (Corning, Corning, NY, USA) and cultured for 6 days with media changes every other day. On day 7 after viral reprogramming, the media was replaced with a 1:1 mixture of PBMC-M and Essential 8 media (E8; Thermo Fisher). The cells were harvested with TrypLE Select Enzyme (Thermo Fisher) and plated in E8 supplemented with 10 µm Y-27632 dihydrochloride (Tocris Bioscience, Bristol, United Kingdom) on plates coated with Geltrex matrix (Thermo Fisher). Colonies were then picked by pipette under a microscope, replated into separate wells of a tissue culture plate, and expanded until passage 10. DNA was then isolated to verify the loss of the Sendai virus and clones were screened via sanger sequencing and karyotyping.

PBMC growth media (PBMC-M) formulation:

- IMDM/F12 (Life Technologies, Frederick, MD, USA)
- 2 mg/mL recombinant human albumin
- 1% v/v chemically defined lipid concentrate (Life Technologies)
- 10 µg/mL recombinant human insulin
- 100 µg/mL recombinant human transferrin

- 15 ng/mL sodium selenite
- 64 µg/mL L-ascorbic acid 2-phosphate
- 450 µM 1- thioglycerol (Sigma-Aldrich)
- 100 ng/mL SCF (Peprotech)
- 10 ng/mL IL3 (Peprotech)
- 2 U/mL EPO (R&D Systems, Minneapolis, MN, USA),
- 40 ng/mL IGF1 (Peprotech)
- 1 µM dexamethasone (Sigma-Aldrich).

*hiPSC-CM differentiation and culture*

All hiPSCs lines were plated onto wells of 6-well tissue culture-treated polystyrene plates coated with 1:200 diluted Geltrex (Life Technologies) in DMEM/F-12, HEPES. For the first 18-22 hours, hiPSCs were maintained in Essential 8 medium (E8) with 10 µM Y-27632 dihydrochloride (Tocris, Bristol, UK). Monolayers of hiPSCs were rinsed with DMEM/F-12 and fed with fresh E8 daily. On the fourth day after plating, when cells had reached about 70-90% confluency, the media was replaced with RPMI 1640 medium supplemented with B-27 supplement minus insulin ("B27-minus", Thermo Fisher Scientific) with 6 µM CHIR-99021 (Selleck Chemicals) to initiate differentiation (day 0 of differentiation). Thereafter, media was changed as follows: day 2, B27-minus ; day 3, B27-minus with 5 µM IWR-1 (Sigma-Aldrich Corp); day 5 and 7, B27-minus; and day 9 and everyother day afterward, RPMI 1640 medium with B-27 supplement with insulin ("B27+"). Spontaneous beating was observed in monolayers starting at day 7 to 9.

Between day 11 and day 13, wells of differentiated hiPSC-CMs were washed with 0.5 mM EDTA and incubated in EDTA for 5-10 minutes at 37 °C. Afterwards, the EDTA was aspirated off and 0.05% Trypsin-EDTA was added for 3-5 minutes at 37°C. Cells were triturated before Defined Trypsin Inhibitor (DTI, Life Technologies) was added to stop digestion. The resultant suspension was centrifuged at 200 g for 5 minutes. After aspirating off the supernatant, the cell pellet was resuspended in B27+ into a homogenous suspension of cells and distributed over 6-well plates or T-75 flasks freshly-coated with 1:200 Geltrex at a surface area ratio of approximately 1:1.5.

On day 14 and day 16, media was replaced with glucose-free DMEM media supplemented with 4 mM L-lactate, NEAA, and glutamax ("lactate medium") to select for and purify cardiomyocytes in culture. After 4 cumulative days in lactate medium, hiPSC-CMs were switched back to B27+ media, and media was replaced every 2-3 days thereafter.

On day 28, cells are passaged as above, using Trypsin-EDTA and Define Trypsin Inhibitor, centrifuged and resuspended in fresh B-27+ media with 10 µM Y-27632 dihydrochloride. Cells in suspension were counted using a Countess automatic cell counter (Invitrogen) and plated at 250,00 cells per $cm^2$ of surface area into tissue culture plates coated with 1:200 geltrex. For imaging and optical mapping experiments, Thermanox plastic coverslips (Thermo Fisher) were placed into 24 or 48-well plates before geltrex coating to allow easy removal of the monolayers at the time of analysis.

*Optical mapping analysis*

Intensity recordings at each pixel were de-noised using a Bayesian $L_1$-regularized global filter and convolved with a 5x5 spatial Gaussian filter. Activation times were defined as the moment where maximum of the derivative of membrane potential (dV/dt) occurs. Histograms of local conduction velocities for each EHS were fitted to a Gaussian curve, and the mean of the curve was defined as the average conduction velocity (CV). Action potential durations measured at 80% and 30% recovery

($APD_{80}$, $APD_{30}$) were determined for all local traces over the recording region for each EHS and fit with Gaussian curves to determine the mean value for each EHS, as described for CV measurements. Peak relative upstroke velocity was calculated by normalizing the action potential traces at each pixel such that it varied between 0 and 1, calculating the differential along the curve and selecting the point in the upstroke where d/dt was maximized. For calcium mapping data, time-to-peak was measured by identifying the time point at which the calcium transient begins to rise and the point at which it reaches its highest signal value and taking the difference. The normalized decay rate (i.e $\tau^{-1}$) was measured by normalizing the calcium transient, modeling the portion of the calcium transient between 30% and full decay as an exponential, and extracting the exponential constant. Spatial heterogeneity of all parameters was measured using the gaussian fits; for each parameter, the standard deviation was divided by the mean to provide relative standard deviation (RSD) of that parameter within the recording region.

*Tyrode's media formulation*

Combine all solutes, warm to 37 ºC and adjust pH to 7.37 using HCl (and NaOH if necessary). Osmolarity should be ~293 mmol/kg

- 5 mM HEPES
- 1 mM magnesium chloride hexahydrate
- 5.4 mM potassium chloride
- 135 mM sodium chloride
- 0.33 mM sodium phosphate
- 1.8 mM calcium chloride
- 5.0 mM glucose
